# Carriage of extended-spectrum beta-lactamase-producing enterobacteriacae among internal medicine patients in Switzerland

**DOI:** 10.1186/2047-2994-2-20

**Published:** 2013-06-12

**Authors:** Janet Pasricha, Thibaud Koessler, Stephan Harbarth, Jacques Schrenzel, Véronique Camus, Gilles Cohen, Arnaud Perrier, Didier Pittet, Anne Iten

**Affiliations:** 1 Infection Control Program, University of Geneva Hospitals and Medical Faculty, 4 Rue Gabrielle-Perret-Gentil, 1211, Geneva 14, Switzerland; 2 Department of General Internal Medicine, University of Geneva Hospitals and Faculty of Medicine, Geneva, Switzerland; 3 Central Laboratory of Bacteriology, University of Geneva Hospitals and Faculty of Medicine, Geneva, Switzerland; 4 Division of Medico-Economic Analysis, University of Geneva Hospitals and Faculty of Medicine, Geneva, Switzerland; 5 WHO Collaborating Centre on Patient Safety, University of Geneva Hospitals and Faculty of Medicine, Geneva, Switzerland; 6 The Jenner Institute, University of Oxford, Oxford, UK

**Keywords:** Extended-spectrum beta-lactamase producing enterobacteraciae, Infection control, Antimicrobial resistance

## Abstract

**Background:**

The incidence of extended-spectrum beta-lactamase producing-enterobacteriacae (ESBL-E) infection is rising worldwide. We aimed to determine the prevalence and nosocomial acquisition rate of ESBL-E as well as the risk factors for ESBL-E carriage and acquisition amongst patients consecutively admitted to 13 internal medicine units at our hospital who were not previously known to be ESBL-E carriers.

**Findings:**

We screened all patients admitted or transferred to internal medicine units for ESBL-E on admission and discharge using rectal swabs. Of 1072 patients screened, 51 (4.8%) were carriers of an ESBL-E at admission. Of 473 patients who underwent admission and discharge screening, 21 (4.4%) acquired an ESBL-E. On multivariate analysis, diabetes mellitus without end-organ complications (OR 2.87 [1.09-7.08]), connective tissue disease (OR 7.22 [1.17-44.59]), and liver failure (OR 8.39 [1.55-45.45]) were independent risk factors for carriage of an ESBL-E upon admission to hospital (area under the ROC curve, 0.68). Receipt of a first- or second-generation cephalosporin (OR 9.25 [2.22-37.82]), intra-hospital transfer (OR 6.68 [1.71-26.06]), and a hospital stay >21 days (OR 25.17 [4.18-151.68]) were associated with acquisition of an ESBL-E during hospitalisation; whilst admission from home was protective (OR 0.16 [0.06-0.39]) on univariate regression. No risk profile with sufficient accuracy to predict previously unknown carriage on admission or acquisition of ESBL-E could be developed using readily available patient information.

**Conclusions:**

ESBL-E carriage is endemic amongst internal medicine patients at our institution. We were unable to develop a clinical risk profile to accurately predict ESBL-E carriage amongst these patients.

## Findings

### Introduction

ESBL-E are found worldwide with rates varying between countries and institutions [1,2]. Infections with ESBL-E are associated with increased morbidity and mortality [3]. Early detection of ESBL-E carriage could allow timely implementation of infection control measures and the appropriate selection of antimicrobials [4]. On-admission surveillance for ESBL-E has been associated with a reduced incidence of ESBL-E infections during hospitalisation [5]. However, universal screening for ESBL-E is time consuming, expensive and unlikely to be feasible [6,7]. Targeted screening based on clinical prediction tools could therefore be useful. The development of such tools requires knowledge of the local epidemiology of ESBL-E [8].

We hypothesized that carriage of ESBL-E would be a significant problem amongst internal medicine patients at our institution and that readily available clinical data could be used to formulate a prediction tool that could accurately predict ESBL-E carriage upon admission to our hospital.

### Setting and methods

The University of Geneva Hospital (HUG) is a 2200-bed hospital providing in- and outpatient care for the Canton of Geneva and surrounding area (~800,000 population). From March to June 2010, all patients >18 years of age consecutively admitted to 13 internal medicine wards at HUG were screened for ESBL-E. Patients underwent admission and discharge rectal swabs performed by ward nurses. Demographic data were collected. Electronic patient records were reviewed retrospectively to obtain data on co-morbid conditions and antibiotic use. This study was approved by the institutional review board as a continuous quality improvement project. No informed consent was therefore required.

Rectal swabs were plated directly onto chromID ESBL medium (bioMérieux, Lyon, France). The presence of ESBL-E was confirmed using the disc diffusion method as described in the 2009 guidelines of the US Clinical and Laboratory Standards Institute (http://www.clsi.org). This study included ESBL- but not AmpC-producing Enterobacteriaceae, which were excluded by phenotypic confirmatory tests. A swab was defined as “on admission” if it had been performed within 48 h of admission to an internal medicine unit. A “discharge swab” was performed within 36 h of discharge. “In-hospital acquisition” was established if patients had an ESBL-E identified on a discharge swab where the admission swab had been negative. Laboratory records at HUG were used to identify patients in whom an ESBL-E had been cultured previously. Patients who had a laboratory sample positive for ESBL-E in the previous six months were identified as previous ESBL-E carriers, and were excluded from the risk factor analysis. This included both clinical isolates and surveillance swabs. In addition, patients whose swabs had not been performed within the pre-specified time frames were excluded from the risk factor analysis. Baseline characteristics of patients who were not captured by the screening programme were compared to patients who were screened using chi squared tests for categorical variables and student t tests for interval variables. Factors potentially associated with previously unknown carriage of ESBL-E on admission and ESBL-E acquisition on discharge were first evaluated using univariate logistic regression. Variables were retained if the *P-*value was <0.2. Multivariate models were then developed with stepwise elimination of variables using likelihood ratio tests to compare each model to the previous one. The performance of the final model was assessed using the area under the receiver operating curve (ROC), using STATA 11.2 (STATA Corp, College Station, TX) for analysis.

## Results

Of 1623 patients admitted to internal medicine, 1072 (66%) underwent admission screening within 48 hours and 39 patients had admission screening performed outside of the 48-hour window. Patients who were not screened at admission were slightly younger (median age, 59.5 vs 62.3 years; *p* = 0.006), more likely to be transferred to internal medicine from another unit rather than directly admitted (4.2% vs 2.4%; *p* = 0.023) and were less likely to suffer from acute renal failure (16.7% vs 23.0%; *p =* 0.028).

Overall, 487 (30%) underwent discharge screening. Patients who failed to have discharge screening performed were younger (median age, 63.1 years vs 66.7 years; *p* = 0.001) and less likely to have chronic obstructive pulmonary disease (11.2% vs 20.2%; *p* = 0.001) and carotid artery stenosis (0.5% vs 2.2%; *p* = 0.027) than those who did undergo discharge screening. There were more males in the group who missed discharge screening (male sex, 60.2% vs 52.1%; *p* = 0.002).

Of 1072 patients who had appropriate admission screening performed, median age was 62.3 years (95% confidence interval, 61.1-63.4) and 56.4% (n = 627) were male. The majority of patients (91.0% [89.2-92.6%]) were admitted to internal medicine directly, whilst 72 (6.7% [5.2-8.1%]) were transferred from the intensive care unit and 26 (2.4% [1.5-3.3%]) were transferred from another hospital unit. Among the 225 patients for whom previous laboratory data were available, 28 (16.0% [11.1-20.8%]) had a laboratory sample that was previously positive for ESBL-E, and were identified as previous ESBL-E carriers. Of 1072 patients screened at admission, 4.8% (51/1072) were identified as ESBL-E carriers. Of 487 patients screened at discharge, 14 were identified as previous ESBL-E carriers and were excluded from the analysis. Of the remaining 473 patients without previously known ESBL-E carriage, 4.4% (21/473) acquired an ESBL-E during their hospital admission (without clinically symptomatic infection). The most commonly identified enterobacteriaceae on screening (admission and discharge swabs combined) were *Escherichia coli* (75.6% [65.9-85.3%]), followed by *Enterobacter cloacae* (5.2% [0.1-10.1]), *Citrobacter freundii* (5.1% [0.1-10.1]), *Morganella morganii* (3.8% [-0.5-8.2]), and *Proteus mirabilis* (2.6% [-1.0-6.2]). Other enterobacteraciae, including *Klebsiella pneumoniae* accounted for 1% or less of isolates.

The results of multivariate logistic regression examining factors potentially associated with previously unknown carriage of ESBL-E on admission/transfer to internal medicine are presented in Table 1. Diabetes mellitus without end-organ complications, connective tissue disease, and liver failure were identified as independent risk factors for ESBL-E carriage upon admission. However, this multivariate model had low predictive accuracy (area under the ROC curve, 0.68).

**Table 1:** **Factors associated with ESBL**-**E carriage amongst 1072 internal medicine patients**, **University of Geneva Hospitals**, **March**-**June 2010**^**1**^; **univariate and multivariate regression analysis**

	**Proportions (n)**		**Univariate regression**		**Multivariate regression**	
**Variable**	**ESBL carriage (n = 51)**	**No ESBL carriage (n = 1021)**	**OR [95% CI]**	***P-*value**	**OR [95% CI]**	***P-*value**
Male gender	54.9 (28)	56.9 (581)	0.92 [0.52-1.62]	0.778		
Transferred from ICU	0	0.6 (6)	0.27 [0.36-1.97]	0.195		
Transferred from other ward (other than the ICU)	2.0 (1)	2.5 (25)	0.80 [0.11-6.00]	0.825		
Patient admitted from home	96.1 (49)	90.7 (925)	2.52 [0.61-10.62]	0.201		
Age						
< =59 y	37.3 (19)	33.6 (343)	1.94 [0.55-6.81]	0.300		
60 -79 y	41.2 (21)	39.5 (403)	1.62 [0.47-5.52]	0.443		
> = 80 y	21.5 (11)	22.1 (226)	1.40 [0.38-5.2]	0.614		
Acute renal failure^2^	20.8 (5)	23.0 (94)	0.87 [0.32-2.40]	0.793		
Chronic renal failure^2^	16.7 (4)	13.6 (55)	1.28 [0.42-3.87]	0.667		
End stage renal failure^2^	4.2 (9)	2.0 (8)	2.16 [0.26-18.04]	0.476		
**Diabetes without complications**^**2**^	**33.3 (8)**	**15.3 (62)**	**2.77 [1.14-6.39]**	**0.033**	**2.87 [1.09-7.08]**	**0.032**
Diabetes mellitus (with complications)^2^	12.5 (3)	10.3 (42)	1.24 [0.35- 4.33]	0.706		
Peripheral vascular disease^2^	8.3 (16)	3.9 (16)	2.21 [0.48-10.25]	0.309		
Chronic obstructive airway disease^2^	20.8 (5)	13.8 (56)	1.64 [0.59-4.58]	0.341		
Dementia^2^	0	1.2 (5)	Omitted			
Stroke^2^	0	1.0 (4)	Omitted			
Cerebral hemorrhage^2^	0	0.5 (2)	Omitted			
Congestive cardiac failure^2^	29.2 (7)	23.7 (96)	1.33 [0.54-3.30]	0.539		
Ischemic heart disease^2^	12.5 (3)	11.1 (45)	1.15 [0.33-4.00]	0.831		
Hematological malignancy^2^	0	5.4 (22)	Omitted			
Carotid artery stenosis^2^	0	1.0 (4)	Omitted			
Parkinson’s disease^2^	0	0.3 (1)	Omitted			
**Connective tissue disease**^**2**^	**8.3 (5)**	**1.0 (4)**	**9.14 [1.58-52.62]**	**0.013**	**7.22 [1.17-44.59]**	**0.033**
**Liver failure**^**2**^	**8.3 (6)**	**1.5 (6)**	**6.06 [1.15-31.77]**	**0.033**	**8.39 [1.55-45.45]**	**0.014**
Respiratory failure^2^	0	0.7 (3)	Omitted			
Solid organ cancer^2^	4.2 (1)	17.0 (69)	0.21 [0.28-1.58]	0.130		
Metastatic cancer^2^	8.3 (2)	8.4 (34)	0.99 [0.22-4.41]	0.994		
Peptic ulcer disease^2^	4.2 (1)	2.7 (11)	1.56 [0.19-12.64]	0.676		
Infection^2^	0	0.9 (4)	Omitted			

1. Positive ESBL swab at admission in cases where the swab was collected within 48 h of admission and patients with prior carriage have been excluded.

*ICU* intensive care unit, *y* years;

2. Denominator adjusted for number of patients in whom data were available.

On univariate regression transfer to internal medicine from another unit and from the intensive care unit were risk factors for nosocomial acquisition of an ESBL-E, whilst admission from home was protective (all *P*-values < 0.01). Other important risk factors for ESBL-E acquisition were: hospital stay >21 days and receipt of a first- or second- generation cephalosporin. These data are presented in detail in Table 2. We did not perform multivariate regression modeling to predict nosocomial acquisition of ESBL-E, due to the small number of patients who acquired ESBL-E.

**Table 2:** **Factors associated with ESBL-E acquisition amongst 473 internal medicine patients, University of Geneva Hospitals, March**-**June 2010**^**1**^; **univariate regression analysis**

	**Proportions (n) or Median [range]**		**Univariate regression**	
**Variable**	**ESBL acquisition (n = 21)**	**No ESBL acquisition (n = 452)**	**OR [95% CI]**	***P-*value**
Male gender	57.1 (12)	51.3 (232)	1.2 [0.51-3.05]	0.603
**Transferred from ICU**	**28.6 (6)**	**8.2 (37)**	**4.48 [1.64-12.25**	**0.003**
**Transferred from other ward (other than the ICU)**	**14.3 (3)**	**2.4 (11)**	**6.69 [1.71-26.06]**	**0.006**
**Patient admitted from home**	**57.1 (12)**	**89.4 (404)**	**0.16 [0.06-0.39]**	**<0.001**
Age (y)	66.9 [30.6-86.8]	64.1 [18.8-99.2]		
Age				
< = 59 y			1.45 [0.16-12.90]	0.737
60 -79 y			1.35 [0.16-11.19]	0.778
> = 80 y			1.89 [0.23-15.99]	0.556
Length of stay (days)	12.1 [3–58]	7.7 [1–54]		
Length of stay				
<7 d			2.69 [0.73-9.95]	0.138
7-14 d			3.09 [0.67-14.23]	0.146
14-21 d			1.5 [0.15-15.13]	0.718
** >21 d**			**25.17 [4.18-151.7]**	**<0.001**
Acute renal failure^2^	45.4 (5)	22.7 (45)	2.83 [0.83-9.71]	0.098
Chronic renal failure^2^	27.2 (3)	14.6 (29)	2.12 [0.55-8.72]	0.268
End stage renal failure^2^	0.0 (0)	3.0 (6)	Omitted	
Diabetes without complications^2^	9.1 (1)	18.4 (38)	0.45 [0.05-3.62]	0.453
Diabetes mellitus (with complications)^2^	9.1 (1)	11.6 (23)	0.76 [0.09-6.22]	0.799
Peripheral vascular disease^2^	0.0 (0)	5.1 (11)	Omitted	
Chronic obstructive airway disease^2^	18.2 (2)	21.2 (42)	0.83 [0.17-3.96]	0.811
Dementia^2^	0.0 (0)	1.0 (2)	Omitted	
Stroke^2^	0.0 (0)	1.0 (2)	Omitted	
Cerebral hemorrhage^2^	0.0 (0)	1.0 (2)	Omitted	
Congestive cardiac failure^2^	9.1 (1)	25.8 (51)	0.29 [0.04-2.30]	0.241
Ischemic heart disease^2^	0.0 (0)	9.6 (19)	Omitted	
Hematological malignancy^2^	0.0 (0)	5.1 (11)	Omitted	
Carotid artery stenosis^2^	0.0 (0)	2.5 (5)	Omitted	
Parkinson’s disease^2^	9.1 (1)	0.5 (1)	19.70 [1.15-338.43]	0.040
Connective tissue disease^2^	0.0 (0)	1.0 (2)	Omitted	
Liver failure^2^	9.1 (1)	1.0 (2)	9.80 [0.82-117.39]	0.072
Respiratory failure^2^	0.0 (0)	0.5 (1)	Omitted	
Solid organ cancer^2^	18.1 (2)	14.1 (28)	1.35 [0.27-6.57]	0.711
Metastatic cancer^2^	0.0 (0)	6.6 (13)	Omitted	
Peptic ulcer disease^2^	9.1 (1)	3.0 (6)	3.20 [0.35-29.18]	0.302
Infection^2^	0.0 (0)	0.5 (1)	Omitted	
Any antibiotic use	42.9 (9)	26.5 (120)	2.08 [0.85-5.04]	0.108
Amoxicillin, flucloxacillin, phenoxymethylpenicillin	14.3 (3)	8.6 (39)	1.76 [0.49-6.25]	0.379
Ceftriaxon	4.8 (1)	1.3 (6)	3.72 [0.43-32.35]	0.234
**Cefazolin, cefuroxime**	**14.3 (3)**	**1.8 (8)**	**9.25 [2.22-37.82]**	**0.002**
Macrolide, tetracycline	0.0 (0)	1.3 (6)	Omitted	
Fluoroquinolone	0.0 (0)	6.0 (27)	Omitted	
Ertapenem, imipenem, meropenem	0.0 (0)	0.2 (1)	Omitted	
Cotrimoxazole	0.0 (0)	1.3 (6)	Omitted	
Vancomycin	4.8 (1)	0.4 (2)	11.34 [0.97-129.31]	0.052
Metronidazole	4.8 (1)	1.8 (8)	2.84 [0.33-23.27]	0.347
Gentamicin	0.0 (0)	0.3 (1)	Omitted	

1. Positive ESBL swab at discharge where admission ESBL-E screening had been negative. Swabs must be collected within 36 h of discharge and patients with prior ESBL-E carriage were excluded.

*ICU* intensive care unit; *y* years, *d* days;

2. Denominator adjusted for number of patients in whom data were available.

## Discussion

Our prevalence of on-admission ESBL-E carriage of 4.8% is similar to other European studies where rates ranging from 2.7 to 30% have been observed [9]. There are no published population-based studies of ESBL-E carriage rates from Switzerland, but the 2010 Swiss national surveillance program (http://www.antibioticresistance.ch) found that amongst hospitalized patients, 5.8% of *E. coli* and 7.1% of *K. pneumoniae* were resistant to third-generation cephalosporins. On-admission carriage rates of 16-27% for ESBL-E have been previously described at our institution when “high risk” groups (patients migrating from, or previously hospitalized in a country with a high prevalence of ESBL-E) were targeted [10].

We cannot account for the low rate of ESBL-producing *K. pneumonia*e found in this study. This finding is surprising, given this organism comprised 32% of the ESBL-E found in previous studies at our institution [10]. A possible explanation could be that previously reported higher rates of ESBL-*Klebsiella* spp were documented in patients transferred from abroad, whereas the current study focused more on community carriers of ESBL-E that could be related to the food reservoir of resistant *E.coli*.

The largest study to examine risk factors for carriage of ESBL-E on admission to hospital is a meta-analysis by Ben-Ami et al., which included 3 studies of on-admission ESBL-E carriage from hospitals in Tel-Aviv and Spain [11]. Male gender, age > 65 years, admission from a long-term care facility, and recent antibiotic use were independent risk factors for ESBL-E on-admission carriage. Despite the fact that the authors had a combined dataset of over 900 ESBL-E-positive patients, the multivariate model was still poorly predictive of ESBL-E carriage. We found that a past history of liver disease, diabetes mellitus, and connective tissue disease were associated with carriage of ESBL-E upon admission. Whilst liver disease has been identified as a risk factor for on-admission ESBL carriage elsewhere [12], diabetes mellitus and connective tissue disease have not. Infections with ESBL-E in patients with severe liver disease are associated with poorer outcomes [13,14]. The mechanisms underlying this association require further elucidation. One possibility is that chronic liver disease may be acting as a surrogate marker for prophylactic fluoroquinolone use against spontaneous bacterial peritonitis — a known risk factor for ESBL-E acquisition [15,16].

Our model was poorly predictive of ESBL-E carriage upon admission to our hospital and this has been found by other studies [11]. Indeed, Ben-Ami et al. found that 20% of patients colonized with ESBL-E at admission had no identifiable risk factors [17]. Similarly, Ruppe et al. studied on-admission characteristics of 500 internal medicine patients and were unable to develop a tool that could effectively predict ESBL-E carriage on admission to their hospital [18].

Few studies have examined the risk factors for acquiring colonization with ESBL-E during hospitalization. Buke et al. found that the presence of a rapidly or ultimately fatal disease on admission (as measured by a high McCabe score) was associated with ESBL-E colonization on day 30 of admission [19]. Duration of urinary catheterization and mechanical ventilation were found to be risk factors for colonization with ESBL-producing *K. pneumoniae* in a Spanish intensive care unit [20]. We found that a prolonged hospital stay and cephalosporin use were associated with acquisition of ESBL-E carriage. These factors have previously been found to be associated with nosocomial infection with ESBL-E [12,21-23].

Our study has several limitations. First, a large proportion of patients failed to have screening performed. Patients who did not have admission screening performed were younger, more likely to be transferred to internal medicine rather than directly admitted, and less likely to have acute renal failure. The increased proportion of transferred rather than directly admitted patients in those who missed admission screening probably reflects that these patients were not perceived as ‘new’ patients by nursing staff making it more likely that screening would be forgotten. It is unclear why there was a smaller proportion of patients with acute renal failure in those who missed screening. As younger patients tend to be less likely to be ESBL-E carriers the failure to capture these patients may have caused the prevalence of ESBL-E carriage in our patients to be overestimated. Patients who failed to have discharge screening performed were younger and more frequently male than patients who did undergo discharge screening. A possible explanation for these differences might be that younger men were more reluctant to undergo rectal swabs. Nursing staff may also have had less opportunity to capture these patients for screening at the point of discharge. The exclusion of these patients may have caused an over-estimate of our ESBL-E acquisition rate given that younger healthier patients would be less likely to have acquired an ESBL-E during their hospital stay. Second, we used rectal surveillance cultures to detect ESBL-E and these have several well-described limitations [24] with a sensitivity ranging from 42-78% [25,26]. We defined ESBL-E acquisition as the detection of ESBL-E on discharge rectal screening where the admission swab had been negative; however, we cannot exclude the possibility that some patients may have had a “falsely negative” admission swab which could occur because of poor collection technique, initial carriage in urine with later transmission to the gastrointestinal tract or low level of colonization at admission with increased bacterial density following antibiotic exposure during hospitalisation [25]. Third, this study was conducted amongst internal medicine patients at a single institution and thus our findings might not be generalizable to other settings and patient populations.

Nevertheless, our study provides valuable information on the prevalence and epidemiology of ESBL-E at this Swiss tertiary care hospital. Our failure to identify a predictive risk profile of previously unknown ESBL-E when using readily available clinical data highlights the difficulties in implementing targeted ESBL-E on-admission screening programs.

## Competing interests

SH has received a peer-reviewed research grant funded by Pfizer, is a member of the speakers’ bureau for bioMérieux, and is a member of the advisory board of Destiny Pharma, BioMérieux and DaVolterra. JS is a consultant for bioMérieux, Biocartis, and Spinomix, and has received research grants and conference support from Abbott, Becton-Dickinson, and Bruker.

## Authors’ contributions

JP performed the data analysis and prepared the manuscript, TK contributed to the study design, data collection and preparation of the manuscript, SH supervised and led the data analysis, contributed to the study design and supervised the manuscript preparation, JS supervised the laboratory work, VC and GC led the data collection, AP, DP, AI designed the study, contributed to the data analysis and manuscript preparation. All authors contributed to and approved the final manuscript.
